# Metasurface Photodetectors

**DOI:** 10.3390/mi12121584

**Published:** 2021-12-20

**Authors:** Jinzhao Li, Junyu Li, Shudao Zhou, Fei Yi

**Affiliations:** 1 School of Optical and Electronic Information, Huazhong University of Science and Technology, Wuhan 430074, China; d201980654@hust.edu.cn; 2 Raytron Technology Co., Ltd., Yantai 264006, China; junyu.li@raytrontek.com; 3 College of Meteorology and Oceanography, National University of Defense Technology, Changsha 410073, China; zhousd70131@sina.com; 4 Wuhan National Laboratory for Optoelectronics (WNLO), Huazhong University of Science and Technology, Wuhan 430074, China

**Keywords:** metasurface, photodetector, multifunctional photodetection

## Abstract

Photodetectors are the essential building blocks of a wide range of optical systems. Typical photodetectors only convert the intensity of light electrical output signals, leaving other electromagnetic parameters, such as the frequencies, phases, and polarization states unresolved. Metasurfaces are arrays of subwavelength structures that can manipulate the amplitude, phase, frequency, and polarization state of light. When combined with photodetectors, metasurfaces can enhance the light-matter interaction at the pixel level and also enable the detector pixels to resolve more electromagnetic parameters. In this paper, we review recent research efforts in merging metasurfaces with photodetectors towards improved detection performances and advanced detection schemes. The impacts of merging metasurfaces with photodetectors, on the architecture of optical systems, and potential applications are also discussed.

## 1. Introduction

Photodetectors, devices that transduce light into electrical signals, are the fundamental building blocks of various optical systems, such as image sensors, detection and ranging (LIDAR), optical transceivers, and so on [1]. The majority of photodetectors can be classified into two broad categories by their detection mechanisms: (1) Photon detectors that transduce the number of photons into electrical signals via photoelectric effect; (2) thermal detectors that transduce the energy of absorbed light into electrical signals via thermoelectric effects [2]. In essence, the electrical signals of both photon and thermal detectors represent the intensity of incoming light. However, these detectors themselves cannot resolve other important electromagnetic parameters of light, such as phase, polarization states, and frequencies. Thus, the detectors usually need to pair with refractive lenses, spectral filters, polarizers, and other separate optical devices to resolve the full properties of light, which shapes the architectures of various optical systems [3].

Metasurfaces, the 2D form of metamaterials, are arrays of subwavelength structures that can take various forms, including metallic or dielectric optical antennas, truncated optical waveguides, or apertures formed in metallic or dielectric films and their multilayer structures [4,5]. Each of the subwavelength structures in a metasurface can independently engineer the local properties of light, including amplitude, phase, frequency, and polarization states. As such, metasurface provides a versatile platform for developing thin-film planar optical devices for resolving light properties. One disruptive advantage of meta-optics, or metasurface-based optical devices, is that they can be produced with the standard mass fabrication processes of the semiconductor chip industry. One lithographic mask is sufficient to integrate multiple functionalities in a single metasurface. This implies that meta-optics and photodetectors can now be fabricated and assembled in the same foundry. The resulting module will be more compact than conventional ones because part, or all, of the bulk optics are replaced by meta-optics. The packaging and optical alignment can also become easier due to the planar nature of metasurfaces [6,7,8].

In this paper, we review recent research efforts in merging metasurfaces with photodetectors towards improved detection performances and advanced detection schemes. We show that, when combined with photodetectors, metasurfaces can enhance the light-matter interaction at the pixel level and enable the detector pixels to resolve more electromagnetic parameters [9,10]. When fully developed, the resulted high-performance multifunctional photodetectors will eventually transform the architecture of the existing sensor modules, making them more compact, lightweight, and cost-effective.

## 2. Concepts

In this section, basic concepts of photon detectors and metasurfaces are reviewed briefly. Limitations of photon detectors were clarified, and the motivating factors of integrating photodetectors with metasurfaces are elucidated.

### 2.1. Photodetectors

**Figure 1:**
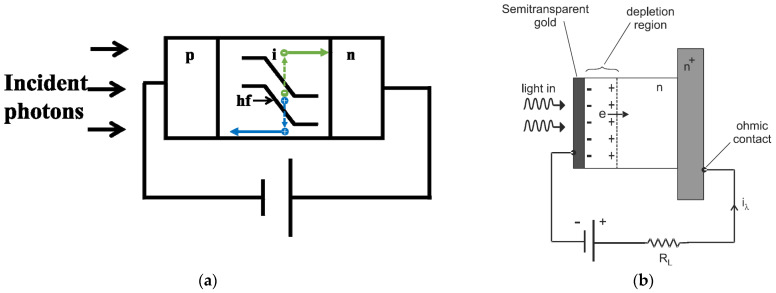

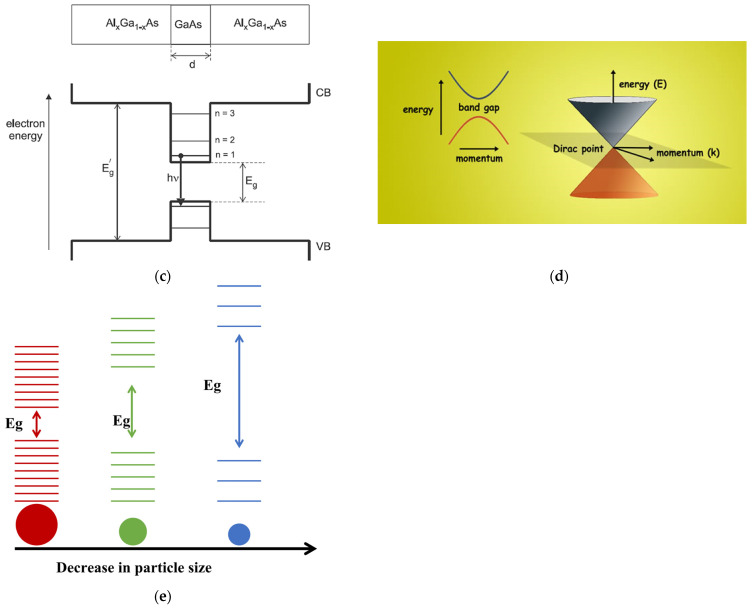
Typical photon detectors: (**a**) PIN photodiode, (**b**) Schottky photodiode, (**c**) quantum well photodetector, (**d**) graphene photodetector, and (**e**) colloidal quantum dots photodetector.

Silicon PIN photodiode (Figure 1a), based on the internal photoelectric effect, is the most commonly used photon detector, in which the semiconductors absorb the incident photons, and the photo-excited electron-hole pairs in the depletion region are separated by the built-in field and collected by the electrodes. However, the low responsivity in the near-infrared (NIR) spectral band restricts its application in light detection and ranging, as well as optical communication.

Schottky photodiodes (Figure 1b) utilize a metal-semiconductor junction with metal-n − n+ or metal-n configuration to separate and collect the photogenerated charge carriers. Incident photons, with energy greater than the Schottky barrier, pass through a semitransparent metallic layer (often gold) and are absorbed in the depletion region in the n-type semiconductor. Charge carriers generated within the depletion region are efficiently swept out by the built-in electric field, giving rise to a photocurrent. The low responsivity of Schottky photodiodes is due to reflection and absorption of light in the metal layer, as well as poor electron injection efficiency [11].

Quantum well infrared photodetector (QWIP) (Figure 1c) is a heterojunction based photodetector, whose energy barrier Eg is determined by the thickness of each layer. However, the intersubband absorption (ISB) in QWIP, at normal incident, is forbidden by polarization selection rule: only the light polarized in the growth direction of the quantum well can cause ISB transitions. Thus, a normal incidence geometry (i.e., light incident normal to the wafer and along the growth direction) is not suitable for photodetection. Thus, various grating structures have been used as the coupler to provide a nonzero polarization component in the epitaxial growth direction [12].

Graphene is also an attractive photodetector material due to its zero-bandgap property (Figure 1d), broadband absorption, and ultrafast photoelectric response. However, graphene photodetector suffers from the weak optical absorption of 2.3% [13]. This motivates the use of on-chip nanostructures to enhance the light matter interaction in graphene photodetectors.

Colloidal quantum dots (CQDs) (Figure 1e) have emerged as a promising nanomaterial for making low-cost, flexible photodetectors with spectral coverage tunable through the quantum confinement effect using complementary metal-oxide-semi-conductor (CMOS) compatible fabrication process. However, CQD photodetectors face the dilemma of low responsivity, resulting from the much thinner active region than the absorption length. Near-field enhancement, using plasmonic nanostructures, is therefore explored as an effective route to increase the light absorption by scattering.

### 2.2. Manipulation of Light by Optical Antennas

Optical antennas are electromagnetic antennas in the optical frequencies. The sizes of optical antennas are usually smaller than the wavelength of light. Thus, optical antennas are subwavelength nanostructures, and the arrays of optical antennas are metasurfaces. Due to the capability of manipulating light at the subwavelength scale, optical antennas and metasurfaces are expected to play significant roles in photodetection by enhancing the light-matter interaction [14] and resolving electromagnetic parameters, such as frequencies and polarization states [15].

The functions of optical antennas in photodetection can be categorized into three schemes [16].

**Figure 2:**
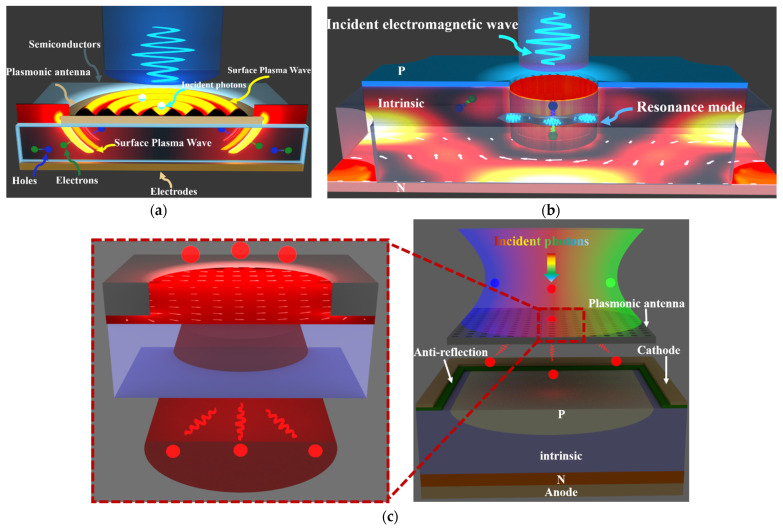
Different functions of optical antennas in photodetection. (**a**) Near-field scattering, causing locally increased absorption, (**b**) coupling the incident light into resonant modes and injecting the photo-excited carriers into the semiconductor, and (**c**) far-field scattering, leading to a prolonged optical path (collect and re-emit photons) [16].

(a) Near field scattering: in this scheme, optical antennas confine the incident light in the vicinity of the nanostructures, enhancing the light-matter interaction and promoting the transduction of photons into electric signals (Figure 2a). Mechanisms for near field scattering include surface plasmon resonance (SPR) and localized surface plasmon resonance (LSPR). SPRs arise from the coupling of electromagnetic fields to the oscillation of the electron plasma in metals, while LSPRs are non-propagating excitations of the conduction electrons in metallic nanostructures coupled to the electromagnetic fields [17].

**Figure 3:**
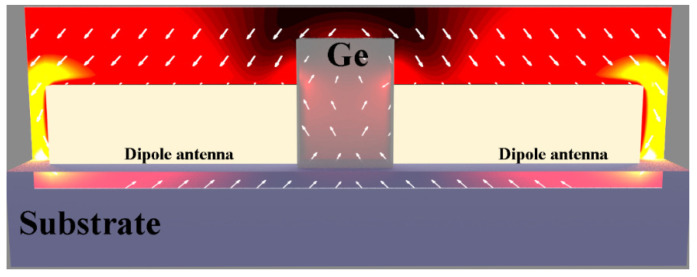
A nanometer-scale germanium photodetector with metal-semiconductor-metal (MSM) configuration is embedded into the gap region of half-wave Hertz dipole antenna. The dipole antenna was used to collect light from a large area and concentrate it into the small subwavelength region of the germanium photodetector [19].

The near field scattering of optical antennas was first applied in near-field optical microscopy because of the large scattering cross-section of plasmonic nanostructures [18]. As shown by Figure 3, by embedding the nanometer-scale germanium photodetector into the gap region of half-wave Hertz dipole antenna, the incident light was concentrated by the dipole antenna into the gap region, where the optical energy was transduced by the photodetector into electric signals [19]. The responsivity improvement in this configuration is attributed to the enhanced interaction between the localized fields and the semiconductor material [20].

(b) Resonant detection: in this scheme, the incident light is coupled to the resonant modes in the nanostructured semiconductors and is absorbed efficiently due to the resonantly enhanced light-matter interaction (Figure 2b).

**Figure 4:**
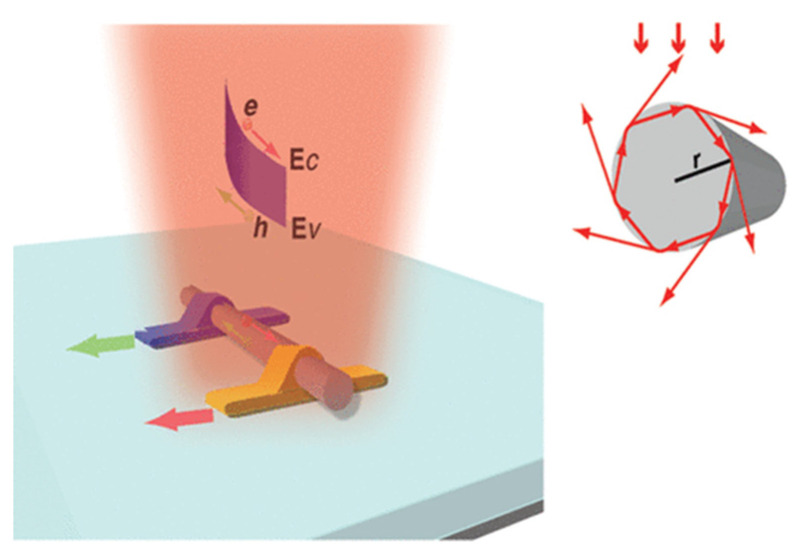
A germanium (Ge) nanowire-based MSM photodetector with asymmetric (one side Ohmic and the other Schottky) metallic contacts.

Resonant detectors provide a compact platform to resolve multiple electromagnetic parameters, including frequencies, polarization states, and the incident angle, based on selective excitation of resonant modes. For example, Mark L. Brongersma proposed a leaky mode resonance-based nanowire photodetector [21]. Sufficiently large nanowires can be thought of as a cylindrical cavity antenna that can trap light in circulating orbits by multiple total internal reflections from the periphery, as illustrated in Figure 4 upper inset. The enhanced photon responsivity is attributed to the coupling of the incident light to leaky mode resonances (LMRs) supported by the nanowires. The resonant modes trap and confine light inside the nanowire, resulting in extended photon lifetime and improved absorption efficiency.

(c) Far field scattering: in this scheme, the metasurfaces (arrays of optical antennas) collect and selectively scatter (or transmit) the incident light into far field, functioning as optical filters, polarizers, or other optical devices (Figure 2c).

**Figure 5:**
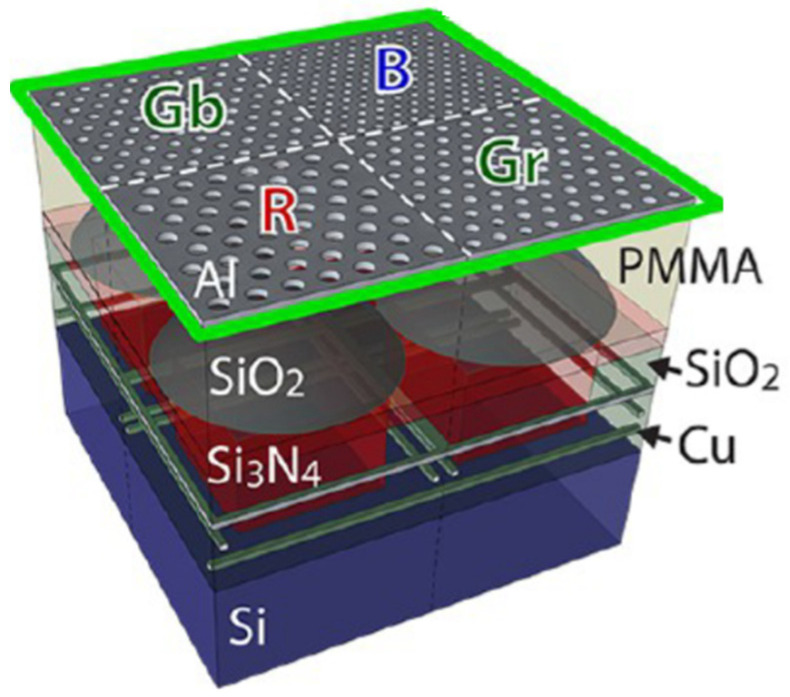
Front-side-illumination CMOS image sensor integrated with RGB plasmonic hole array filters in Bayer mosaic layout.

An example of this scheme is to use the extraordinary optical transmissions [22] through subwavelength holes, perforated in metallic films, to control the spectrum and polarization states of the transmitted light. Since the fabrication of such metasurface filters are CMOS compatible, they can be integrated with the detector pixels of image sensors to control the spectral responsivity and polarization responsivity of each detector pixel. As shown in Figure 5, Stanley P. Burgos integrated perforated Aluminum film on polymethyl methacrylate (PMMA) planarization spacer above commercial image sensor [23]. The incident light coupled to surface plasmons in reflection gratings and was scattered to the photodetector, by the coupling of surface plasmons, on two sides of perforated Aluminum film.

In the following sections, we will provide a more detailed review on recent research efforts in merging metasurfaces with photodetectors towards improved detection performances and advanced detection schemes.

## 3. Near-Field Scattering

### 3.1. SPRs Assisted QWIP

**Figure 6:**
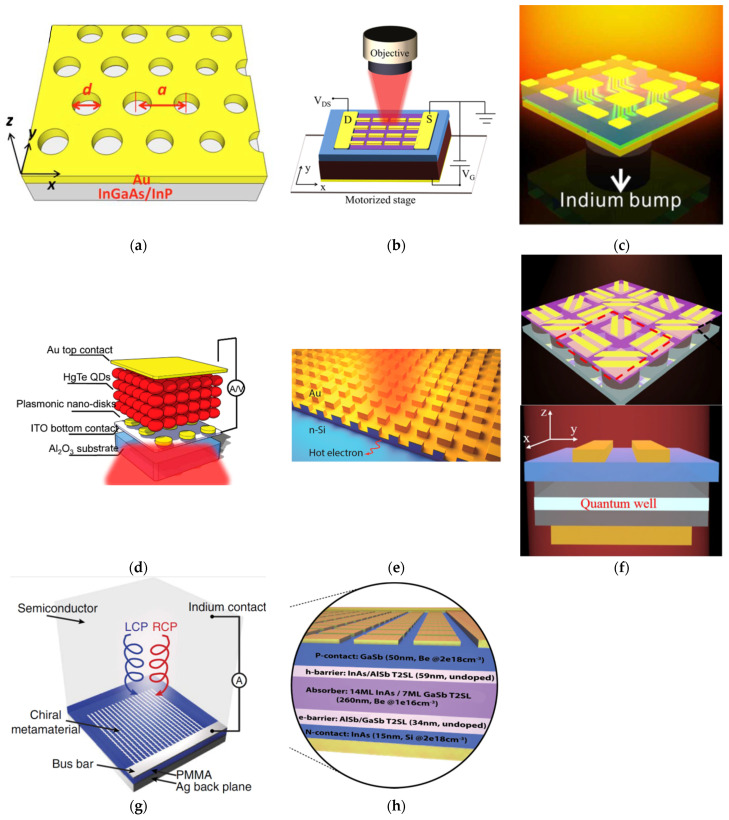
Examples of metasurface integrated photodetectors based on near field scattering: (**a**) SPRs assisted QWIP. (**b**) Plasmonic nanoantenna integrated Graphene photodetector. (**c**) LSPRs assisted QWIP FPA. (**d**) LSPRs assisted HgTe colloidal quantum dot photodetector. (**e**) Metamaterial perfect absorber Schottky photodetector. (**f**) LSPRs assisted linear polarization QWIP. (**g**) Chiral Schottky photodetectors. (**h**) Multi-spectral Type II Superlattice (T2SL) photodetector.

Surface plasmon polaritons have a strong electric component |Ez| perpendicular to the metal-dielectric interface [17], which can be applied to enhance the responsivity of the quantum well infrared photodetector. Wei Wu et al. demonstrated a normal-incident QWIP, strongly coupled with surface plasmon modes (Figure 6a). A periodic hole array perforated in gold film was integrated with In_0.53_Ga_0.47_As/InP QWIP to convert normal-incident electromagnetic waves into surface plasmon waves excite the intersubband transition of carriers in the quantum wells [24]. In this configuration, strong surface plasmon waves are generated in the active region between the holes, and the electric field component |E_z_| is greatly enhanced because of the surface plasmon generation. A large |E_z_| component almost covers the entire lattice period in the active region, and the |E_z_| intensity is still very strong even at 800 nm below Au/semiconductor interface. Therefore, most of the quantum well active region can effectively receive and absorb the electromagnetic waves with a considerable |E_z_| component. The peak responsivity reaches a value as high as ~7 A/W at 8.06 μm, and the corresponding detectivity is calculated to be ~7.4 × 10^10^ cm Hz^1/2^/W. Due to the spectral selectivity of the Au holes arrays, the full width at half maximum (FWHM) of the detector’s spectral response is 0.84 μm, which is almost half of the standard In_0.53_Ga_0.47_As/InP bound-to-continuum QWIP device.

### 3.2. Plasmonic Nanoantenna Integrated Graphene Photodetector

Plasmonic nanoantennas can squeeze the incident light into a volume smaller than the diffraction limit via LSPR, resulting in strongly localized fields at the metal-semiconductor interface [17]. Yu Yao et al. presented an antenna-assisted graphene detector design, where optical antennas are used as both light-harvesting components and electrodes to simultaneously enhance light absorption and carrier collection efficiency (Figure 6b). The antenna-assisted graphene detectors are composed of end-to-end coupled antennas on a graphene sheet. Light incident, from free space, is tightly concentrated into the near-field in the nanogaps between antennas (gap size 100 nm), which can greatly enhance the light-graphene interaction and thus increase light absorption in graphene. Correspondingly, the detector responsivity is enhanced by more than 200 times, to 0.4 V/2 W at λ = 4.45 μm, compared to devices without antennas (<2 mV/W) [25].

### 3.3. LSPRs Assisted QWIP FPA

Since the optical properties of LSPR are neither influenced by the decreasing number of period [26], nor by the increasing angle of incident, it is better suited than SPRs for pixel-level integration with photodetectors. Jing et al. demonstrated a plasmonic microcavity infrared photodetector (PMC-QWIP) with a single quantum well integrated between the top layer of gold nanoantenna array and the bottom layer of gold backplate (Figure 6c). Greater than one order of magnitude enhancement of the peak responsivity (from 0.17 A/W of standard QWIP to 1.8 A/W of PMC-QWIP) has been observed [27]. The significant improvement originates from the highly confined optical mode in the cavity, leading to a strong coupling between photons and the quantum well, which results in the enhanced photoelectric conversion process. Such strong coupling from the localized surface plasmon mode inside the cavity is independent of incident angles, offering a unique solution to high-performance focal plane array (FPA) devices.

### 3.4. LSPRs Assisted HgTe Colloidal Quantum Dot Photodetector

Tang et al. incorporated the plasmonic cavity scheme to improve the responsivity of HgTe colloidal quantum dot photodetector with a thickness of λ/10 (Figure 6d). The configuration of the detector includes a sapphire substrate, an ITO layer as the lower electrical contact, a plasmonic disks array, a thin CQD layer, and a gold layer as both the optical reflector and the upper electrical contact [28]. The vertically stacked configuration creates an optical resonant cavity and enhances the absorption as the incident light bounces between the top contact and the plasmonic disk array. The low-temperature responsivity was increased two- to three-fold, up to 1.62 A/W, with a corresponding external quantum efficiency (EQE) of 45% at 4.5 μm. The enhanced detectors retained background-limited performance with a detectivity of ~4 × 10^11^ cm Hz^1/2^/W. Tang’s work provides a strategy to improve quantum efficiency of Mid-Wave Infrared (MWIR) detector with the active region thinner than absorption length [29].

### 3.5. Metamaterial Perfect Absorber (MPA) Schottky Photodetector

Li et al. proposed a strategy to enhance the photoresponsivity of Schottky photodetector using MPA (Figure 6e). A polarization-dependent MPA, based on 1D stripe resonators, and a polarization-independent MPA, based on 2D square resonators, have been demonstrated. The MPAs have broadband and near-unity absorption. The MPA architecture is integrated with n-type Silicon (n-Si) to realize Schottky photodiode based hot-electron photodetectors, with high photoresponsivity in the near-infrared region, well below the Si bandgap energy. Hot electrons, generated from the decay of LSPRs, have a higher probability of injecting to the Schottky junction due to the ultrathin plasmonic structure. The MPA-based Schottky photodetector reaches a broadband responsivity larger than 1.8 mA/W from 1200–1500 nm [30].

### 3.6. Plasmonic Antenna Integrated Stokes Polarimeter and Multi-Spectral Photodetector

Zhou et al. demonstrated an ultra-compact Stokes polarimeter based on a pixel-level QWIP integrated with plasmonic microcavity (PMC) with 1D gratings (Figure 6f). The materials in the PMC-QWIP pixel, from bottom to top, are: a Ti (50 nm)/Au (300 nm) reflection layer, a 200 nm n-doped GaAs top contact layer, a 207 nm single quantum well layer, a 200 nm n-doped GaAs bottom contact layer, a 300 nm etch stop layer, and a Ti (50 nm)/Au (150 nm) grating layer. The Au gratings improve the coupling efficiency of TM polarized light to QWIP through LSPR and reflect TE polarized light, and a polarization extinction ratio of 136 is reached [31]. By constructing a super pixel, with its four subpixels’ gratings oriented at 0°, 45°, 90°, and 135°, respectively, the first three elements of the Stokes vector can be simultaneously resolved.

To detect circularly polarized light, Li et al. designed a chiral metamaterial, consisting of a periodic array of ‘Z’-shaped silver (Ag) antennas on top of a PMMA spacer and an optically thick Ag backplate (Figure 6g) [32]. The periodic array of silver antennas also allows for electrical connection. The chiral metamaterial is fabricated on an n-type Silicon wafer, with the silver antennas in direct contact with the Silicon substrate, forming a Schottky barrier. Light is incident on the frontside of the Si wafer, transmitting to the backside, where the chiral metamaterial absorbs photons of a particular handedness, generating electrons within the metal at higher energy states. The energetic electrons (or hot electrons), with energy higher than the Schottky barrier, can emit over the Metal-semiconductor interface, leading to a detectable current. The compact chirality selective Schottky photodetector achieves a responsivity of 1.5 mA/W and a polarization discrimination ratio of 3.4.

Montoya et al., demonstrated that, by embedding an ultrathin (λ/15) InAs/GaSb detector region, non-active contact layers, and unipolar barrier layers between the top layer of gold nanoantenna array and the bottom layer of gold backplate, the incident IR radiation can be strongly focused into the active region of the photodetector, and thus, the signal to-noise ratio can be dramatically increased at any desired wavelength (Figure 6h) [33]. At T = 77 K, the quantum was increased from 6.1% to 18.6% (3.1×) at a peak operating wavelength of 6.14 μm and from 4.1% to 14.7% (3.6×) at a peak operating wavelength of 7.13 μm. A specific detectivity at 6.14 μm of 6.06 × 10^10^ cm Hz^1/2^/W for the MWIR design and a value of 5.60 × 10^10^ cm Hz^1/2^/W at 7.13 µm is achieved. The enhanced quantum efficiency is empowered by several key features: (1) reducing the electrical volume, which could reduce the detector dark current down to a fraction of that for a conventional photodetector, (2) a deep sub-wavelength metamaterial pattern array on a pixel significantly improves photodetector absorption efficiency, and (3) spectral filtering is achieved for any desired wavelength with a single photolithography step. Through this effort, an ultra-thin multispectral infrared metamaterial detector can achieve higher frame rates, increased sensitivity, and multicolor detection.

Section discussion: This section elucidates that the near-field scattering scheme improves the responsivity by increasing local density of electromagnetic state and resolves more electromagnetic parameters by frequency or polarization selective local interaction. Near field scattering is preferable to improve the responsivity of the photodetector by integrating plasmonic nanoantennas. However, the non-negligible Ohmic loss in metallic nanoantennas restricts further improvement of the responsivity, which can be reduced by replacing Nobel metals with a highly doped semiconductor [34]. Near field scattering also enables isotropic photodetectors (quantum well photodetector) to resolve linear polarization states with great extinction ratio. By integrating spectrally selective plasmonic nanoantennas onto the photosensitive region, metasurfaces can also convert a photodetector array into a light-weight multicolor imaging system.

## 4. Resonant Detection

### 4.1. Resonant Cavity-Enhanced Photodetector

**Figure 7:**
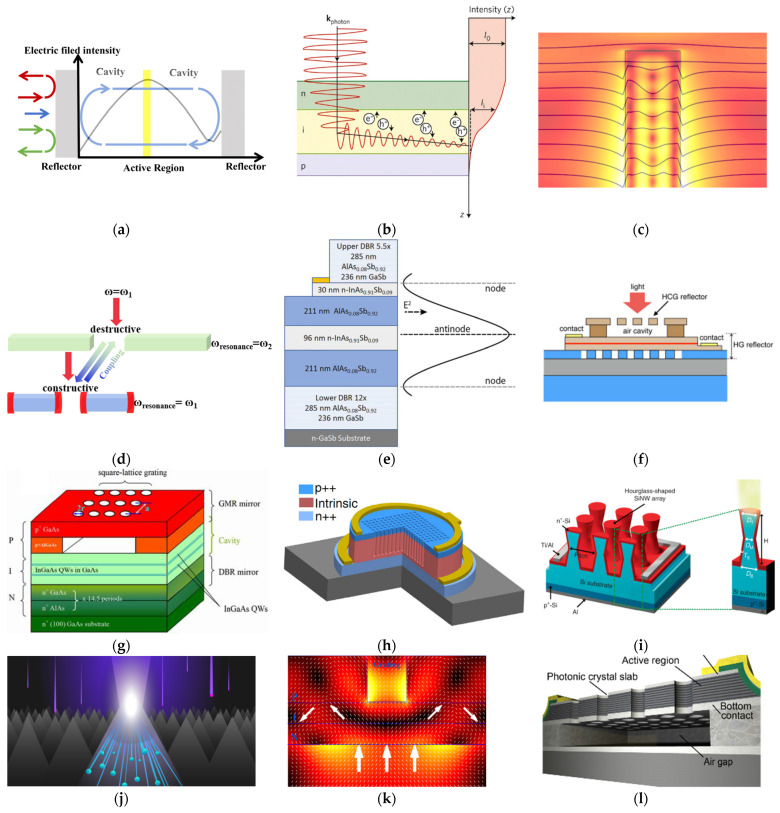

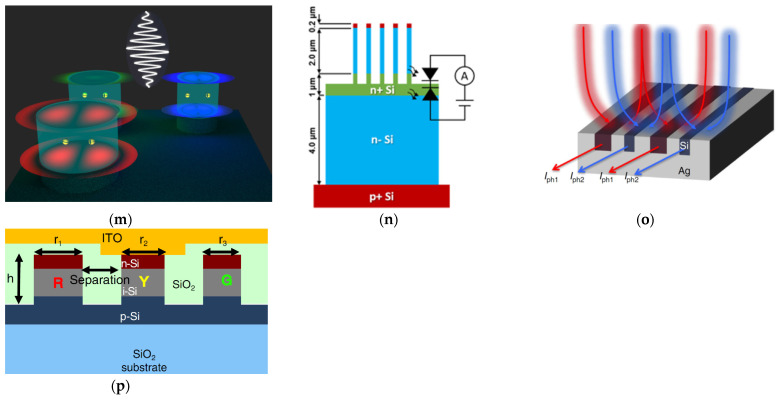
Examples of metasurface assisted resonant photodetectors. (**a**) The basic scheme of resonant cavity-enhanced photodetectors. (**b**) Basic scheme of photo-trapping microstructures assisted photodetector. (**c**) Basic scheme of nanowire waveguide photodetector. (**d**) Basic scheme of Anti-Hermitian nanowire system. (**e**) DBR based RCE-PD. (**f**) HCG-R based RCE-PD. (**g**) PCS-R based multi-wavelength RCE-PD. (**h**) 2D hole array assisted SOI photodetector. (**i**) Whispering gallery mode based hourglass Silicon nanowire photodetector. (**j**) Black Silicon photodetector. (**k**) Guided mode resonance-based InGaAs photodetector. (**l**) Photonic crystal slab-based MQW photodetector. (**m**) Silicon nanowire photodetector. (**n**) Vertically stacked photodetector. (**o**) Anti-Hermitian nanowire photodetectors. (**p**) Anti Hermitian nanorod color sensor.

As shown in Figure 7a, resonant cavity-enhanced photodetector (RCE-PD) is formed by integrating the active absorption region into a resonant cavity composed of top and bottom mirrors [35]. The resonant cavity enhances the responsivity at the resonant wavelength by incorporating the multiple pass detection scheme. Compared with the bulk photodetector, RCE-PD has the advantage of higher responsivity at the resonance wavelength, lower dark current, higher response speed, wider tunability, and narrow spectral response [36]. The optional reflectors of Fabry–Pérot resonant cavity include distributed Bragg reflector (DBR), high contrast grating reflector (HCG-R), photonic crystal slab reflector (PCS-R), and hybrid grating reflector [37]. Craig et al. demonstrated that, by placing 96 nm of InAs_0.91_Sb_0.09_ at the antinode of AlAs_0.08_Sb_0.92_/GaSb DBR based-Fabry–Pérot resonant cavity (Figure 7e), a peak spectral efficiency of 70% was achieved at 3.72 μm [38]. With the narrow bandwidth of 44 nm and the wide block region, the DBR based RCE-PD is suitable for detection of narrow gas absorption features [36]. Compared to DBR, PCS-R and HCG-R make easy fabrication and a highly reflective dielectric mirror achievable.

Learkthanakhachon et al. demonstrated a hybrid RCE-PD structure, in which a 12 nm InGaAs layer is integrated inside the resonant cavity comprising a Silicon HCG-R, an InP hybrid grating reflector, and an air cavity between them (Figure 7f). A narrowband response with FWHM of 3.2 nm at λ = 1548 nm was achieved [39].

Lai et al. demonstrated an RCE-PD structure consisting of a bottom GaAs/AlAs DBR, a cavity with InGaAs/GaAs multiple quantum wells (MQWs) for light absorption and a top mirror of sub-wavelength grating (Figure 7g). With a single layer serving as a grating and also a waveguide, the sub-wavelength grating exhibits wide-band high-reflectivity based on the guided-mode resonance (GMR) effect [40]. Benefiting from its much thinner thickness and flexibility during device processing, the reflector using GMR effect has been implemented in many photonic devices instead of conventional DBR mirror. By changing the fill factor of the GMR grating, the effective cavity length of RCE-PDs can be varied, so the resonant wavelength can be selected post growth.

### 4.2. Photon-Trapping Microstructures Assisted Photodetector

As shown in Figure 7b, photon-trapping microstructures extend the photon lifetime inside the active region through whispering gallery mode, total internal reflection, photonic crystal slab lateral mode, gradient refractive index, and guided mode resonance.

Gao et al. introduced 2D nanohole array into SOI (Silicon on Insulator) planar Silicon photodetector (Figure 7h). The nanohole arrays couple normally incident electromagnetic waves into an ensemble of lateral collective modes propagating along the active region, extending the absorption length by 13 times. The nanohole array assisted Silicon photodetectors achieve an EQE of 50% and an impulse response time of 30 ps at 850 nm [41], which is suitable for high-speed optical communication.

Kim et al. developed Silicon photodetectors (Si PDs) based on a hourglass-shaped nanowire structure, with improved photoresponse in the NIR-SWIR (Short Wavelength Infrared) region. As shown in Figure 7i, the upper, inverted nanocone of the nanowires increases absorption probability by extending the dwell time of NIR-SWIR photons via the generation of whispering-gallery-mode (WGM) resonances, whereas the lower nanocone, with its low reflectance, reabsorbs the light incident from surrounding nanowires. When integrated with Si PD, the hourglass-shaped nanowires tightly confined the incident light via WGM resonances [42,43] and improves the responsivity to 0.59 A/W at 1000 nm, which is close to the responsivity of commercial InGaAs photodetectors [44].

SIONYX improved the responsivity of the backside illuminated Silicon image sensor in NIR by integrating a micro and nano texturing technology with a low noise CMOS image sensor design (Figure 7j). Single crystal Silicon was processed by an ultrafast laser in an SF6 atmosphere. Conical Silicon spikes generated on surfaces reduce optical reflection through gradient index and trap the photons inside cavities between spikes [45]. Furthermore, Huang et al. reported that, by introducing rapid thermal annealing and hydrogenated surface passivation to elevate the broad-bandgap responsivity and signal to noise ratio and to suppress the dark current, the narrower bandgap of Sulphur doped Silicon can reach a specific detectivity of 1.22 × 10^14^ cm Hz^1/2^/W at 1080 nm [46].

Verdun et al. demonstrated a full-dielectric guided mode resonant photodiode (Figure 7k). The device consists of an InP/InGaAs/InP PIN heterojunction containing an active layer as thin as 90 nm on top of a subwavelength lamellar grating and a gold mirror. The benzocyclobutene (BCB) subwavelength grating is implemented at the backside of the detector, so all the nanofabrication steps are prior to the transfer of detector layers, which makes the fabrication process compatible with the indium bonding technique for FPA production. The subwavelength grating couples the normally incident light into waveguide-mode propagating laterally along the active region [47]. It was designed to enhance the electric field across a broader bandwidth than RCE-PD, by excitation of several optical resonances. Near perfect collection of the photo-carriers and EQE, up to 71%, was observed around 1.55 μm [48,49,50].

Kalchmair et al. demonstrated a quantum well infrared photodetector, which is fabricated as a photonic crystal slab (PCS) resonator (Figure 7l). The quasi-TEM modes in the PCS have electric field component perpendicular to QWIP and strong field confinement inside the slab [51]. The strongest resonance of the PCS is designed to coincide with the absorption peak frequency at 7.6 µm of the QWIP. The strong resonant absorption enhancement yields a detectivity enhanced, by 20 times, to 10^11^ cm Hz^1/2^/W. This enhancement is a combined effect of increased responsivity and noise current reduction [52].

### 4.3. Nanowire Waveguide Photodetector

Conventional color sensors are formed by integrating pigment-type filters over a photodetector [53,54]. Crozier et al. proposed that vertical silicon nanowire (SiNW) PIN photodetectors, whose spectral responsivities are controlled by nanowire radius (Figure 7c). The spectral sensitivity of SiNW is related to resonant absorption; incident photons couple to transverse resonance mode and propagate along SiNW [55], offering high absorption efficiency and radius-dependent spectral responsivity. Color imaging experiments were performed using four SiNW arrays (Figure 7m), and the coincidence between pictures obtained from SiNW arrays and conventional camera proves the potentiality of SiNW in color imaging [56].

Filter-free SiNW photodetector is an ultra-compact platform for multi-spectral imaging, in which color pixels are defined through a single lithography step. Based on this platform, Crozier et al. further demonstrated a chip containing 24 pixels, each comprising a Silicon nanowire array photodetector formed above a planar photodetector (Figure 7n). The SiNWs are structurally colored, enabling each pixel to combine wavelength selectivity and photodetection functions in the same chip. The reconstruction of the spectrum, of an unknown light source impinging upon the chip, is achieved by an algorithm that takes as its inputs the measured photocurrents from the pixels and a library of their responsivity spectra [57].

### 4.4. Anti-Hermitian Coupling Photodetector

Anti-Hermitian (AH) coupling introduces a constructive interference among one excitation pathway and destructive interference among other excitation pathways, through which resonators, packed closely, can be excited individually [58]. Based on this concept, Zhang et al., demonstrated the selective excitation of individual plasmonic antennas closely packed within only λ/15 in the Anti Hermitian coupling system (Figure 7d). 

Mark L. Brongersma et al. proposed the Anti-Hermitian photodetector for dual-band photodetection: polycrystalline-silicon (poly-Si) nanobeams with different width are entrenched in an Ag film laterally alternatively. The incident light funnels into corresponding nanowire spectra separately. The silver fins suppress the near field coupling between nanowires, and the scattered fields have a phase difference of π, inferring the near-unity absorption, low energy leakage, and high spectral sorting efficiency. The Anti-Hermitian coupling photodetector sorts and detects photons with a bandwidth of 30 nm [59].

Zhang et al., further demonstrated sub-wavelength scale color pixels in a CMOS compatible platform based on Anti-Hermitian metasurfaces (Figure 7p) [60]. The AH Silicon metasurfaces, with two-dimensional arrays of three differently sized nanocylinders, are coupled with a shallow PIN junction for efficient carrier transport and electrical readout. By carefully controlling the size and separation of Silicon nanocylinders, visible light can be selectively absorbed in multiple color channels with negligible diffraction, leading to spectrally pure absorption profiles of neighboring sub-pixels. The demonstrated metasurface color sensors can sort three colors over a 100 nm bandwidth in the visible regime. 

Section discussion: the resonant modes in nanostructured semiconductors can enhance the responsivity and resolve multiple electromagnetic parameters. Photon-trapping microstructures assisted Silicon photodetectors in finding application in LIDAR because of its high optical absorption efficiency, at 940 nm, and large bandwidth. However, photo-excited carriers quench easily because of the surface-charge recombination on the surface of nanostructured semiconductor, and careful passivation is necessary to achieve high detectivity. Si nanowire photodetectors and Anti-Hermitian photodetectors have the advantage of filter free photon sorting capability, reduced optical crosstalk and smaller pixel size, which is suitable for miniaturized spectrometer. However, their spectral responsivities are different from the transmittance of commercial RGB filter, which restricts their application in a full color image system.

## 5. Far-Field Scattering

### 5.1. Plasmonic Color Filter for Image Sensor

**Figure 8:**
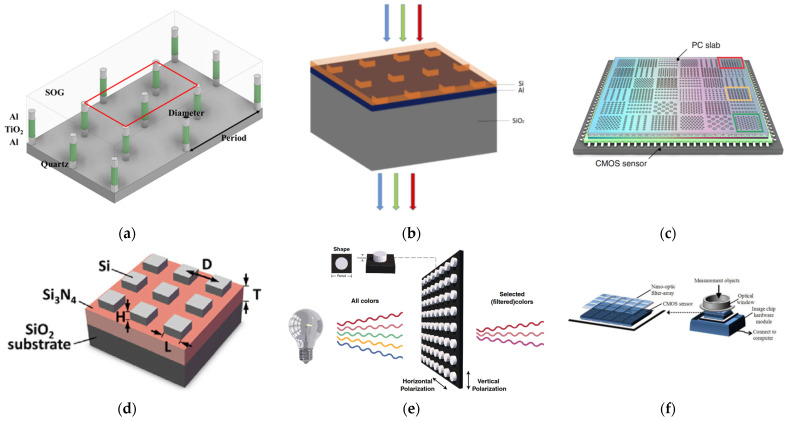
Examples of photodetection assisted by metasurface filters. (**a**) Plasmonic color filters for image sensors. (**b**) Random plasmonic filter-based spectrometer. (**c**) Broadband filter-based spectrometer. (**d**) Broadband encoding stochastic filter-based spectrometer. (**e**) Inverse design of specific filter using deep learning. (**f**) Nano-spectrometer “Apollo”.

Multiple lithography and alignment steps are required for integrating pigment-type color filters over image sensor [61]. The transmittance of plasmonic filters can be tuned by changing the geometries of holes perforated in metallic films, which is preferable in multicolor imaging. It is reported that the filtering functionality of plasmonic filters maintains with a decreasing number of periods and random defect densities. Suppressed spatial crosstalk and angle-independent spectral filtering are critical for micrometer-sized optical filters [62,63], which could be achieved using plasmonic color filters. Plasmonic filters are prime candidates for specific spectral filters, such as CMY filters [64], which transmit twice as much light than RGB filter. He et al. presented subtractive CMY color filters made of Al-TiO_2_-Al nanorods covered by spin on glass (SOG) (Figure 8a). Nanorods absorb one of the RGB lights based on surface plasmon resonance, and the remaining is transmitted through the filter [65].

### 5.2. Coded Optical Filter for Hyperspectral Imaging

Benefiting from the development of image sensors and algorithms, snapshot spectrometer and hyperspectral imager are soon available in the customer market [66]. The filter-based digital spectrometer has the advantage of low cost and portability. Compared with narrowband filters, broadband filters are more appropriate for computational spectrometer [67] because of higher through-output, lower noise, lower cost, lower sampling rate, and more precise construction [68,69,70].

Chen et al. proposed a more accurate spectral reconstruction by making random linear broadband measurements of the spectrum via a nanostructured multispectral filter array (MSFA) than narrow band measurements (Figure 8b) [71,72]. The MSFA consist perforated Aluminum film on SiO_2_ substrate cover by 100 nm Silicon upper cladding. Random transmission filters, based on surface plasmon resonance, have the advantage of random sampling, which reconstructs the spectrum the signal more efficiently. From these random linear measurements of MSFA, the multispectral image is recovered by an optimal reconstruction algorithm that jointly exploits both spectral and spatial sparsity.

Zhu et al. proposed a compact spectrometer formed by an array of photodetectors (Figure 8c), each of which has a unique responsivity with rich spectral features, created by complex optical interference in Silicon on sapphire photonic crystal slabs, positioned immediately on top of the photodetector pixels [73]. Light incident from free-space can couple to lateral propagation modes, where the periodic nanostructures allow light to bounce back and forth many times. The effect of path enhancement in PCS spreads over a broader spectral range and creates a transmission spectrum with rich spectral features, including sharp peaks, due to guided resonances, broad background variation by Fabry–Perot resonance, and irregular line shapes due to Fano interference. A series of narrow-band spectra, with bandwidths of about 1.4 nm and wavelengths varying from 550 to 750 nm with a 1 nm step, generated by a monochromator, was reconstructed by the spectrometer using 36 different photonic crystal structures.

Compressed sensing with broadband encoding stochastic filters (BEST) plays an important role in hyperspectral imaging, which alleviates the conflict between spectral resolution and spatial resolution. Hao et al. reconstructed the spectrum from 400 nm–700 nm, with a bandwidth of 2 nm using four BEST filters (Figure 8d) [74]. BEST includes Silicon nanoblocks above Si_3_N_4_ thin film on SiO_2_ substrate. Optimal BEST filters are given by parameter constrained encoder and decoder method, and metasurface filters are derived from training deep neural networks.

Xiao Han et al. demonstrates that encoded filters capable of filtering light of specific wavelength can be achieved via inverse design by varying planar geometric shape of metasurface, which is an overwhelming advantage over multilayer thin-film filters (Figure 8e) [75]. As shown in Figure 8e, the physical structures of customized metasurface spectral filtering consists 2D pattern poly-Si on Silica Substrate. The pattern of Silicon metasurface is optimized using rigorous coupled-wave analysis (RCWA) and adversarial network to achieve the desired transmittance spectrum. The error rate of transmittance spectrum of optimized and the desired spectrum is 5%, which confirms the potentiality in designing customized spectrum filters.

Byung Il Choi et al. demonstrated a miniaturized spectrometer that measures light in the spectral range of 340–1010 nm by integrating a 32 × 32 plasmonic filter array over a regular CMOS image sensor (Figure 8f) [76]. The plasmonic filters are realized by introducing nanoscale structures on metal films, and the transmission wavelength can be controlled only by the lateral structures on a single layer. While the transmission function of individual spectral sensor is broad, and cannot resolve the spectral component of a particular wavelength, a sensor array of multiple such spectral sensors make it possible a filter-based spectrometers in a chip-scale size. Tikhonov regularization and regularization parameter selection were introduced to solve the spectrum reconstruction problem. Nano-spectrometer “Apollo”, developed by the nano λ company, is the smallest plasmonic filter based commercial snapshot spectrometer, which have bright prospect in Internet-of-Things.

Section discussion: Plasmonic RGB filters have lower transmission efficiency than pigment-type filters due to metals’ intrinsic loss. On the other side, plasmonic CMY filters, which are more transmissive than RGB filters, are promising for low-light imaging applications. Metasurface filters are also useful for constructing coded filters for spectrum reconstruction because of the large degree of freedom and planar nature. However, high-resolution pattern generation technologies, such as electron beam lithography (EBL), nanoimprint lithography (NIL), and deep ultraviolet lithography (DUV), are indispensable to fabricate metasurfaces for applications in visible bands.

## 6. Discussion

To sum up, metasurfaces have been used to improve the detectivities of a variety of photodetectors by enhancing the light-matter interaction at the pixel level, and they also enable the detector pixels to resolve more electromagnetic parameters such as frequencies and polarization states. The planarity of metasurfaces allows for fabrication routes directly in line with conventional processes of the mature integrated circuit industry. The technology required to mass-produce metasurfaces dates back to the early 1990s, when the critical dimensions of semiconductor manufacturing became smaller than the wavelength of visible light. This provides the possibility of unifying the sensor manufacturing and the making of optical components such as spectral filters, polarizers, and lenses, with the same technology used to make computer chips [6]. We therefore envision a future of sensor modules based on metasurfaces with vastly improved Size, Weight, and Power (SWaP) characteristics.
